# Can volunteer medical visit companions support older adults in the United States?

**DOI:** 10.1186/s12877-021-02162-5

**Published:** 2021-04-16

**Authors:** Orla C. Sheehan, Marcela D. Blinka, David L. Roth

**Affiliations:** Center on Aging and Health, Division of Geriatric Medicine and Gerontology, Johns Hopkins University School of Medicine, 2024 E. Monument Street, Suite 2-700, Baltimore, MD 21205 USA

**Keywords:** Medical visit companion, Volunteer, Isolated older adults

## Abstract

**Background:**

Older adults are encouraged to use Medical Visit Companions (MVCs) for routine medical encounters; however, many vulnerable older adults attend alone or fail to attend. In the absence of available family or friends, community volunteers could potentially fill this gap. We aimed to understand the role and acceptability of volunteer MVCs accompanying older adults to medical visits and explore potential barriers and facilitators of increasing MVC availability and expanding roles beyond transportation.

**Methods:**

Two moderators conducted 4 focus groups with 29 volunteers grouped by whether they provided (*n* = 15) or received (*n* = 14) rides to medical visits. All were members of Partners In Care (PIC), a community organization in Maryland, United States which offers a range of programs and services that support the independence of older adults including the provision of volunteer MVCs. Participants were asked to discuss why they were involved with PIC, and to describe their experiences with providing or receiving companionship during medical visits. Inductive thematic analysis was used to explore the views and experiences of participants, particularly around the roles played by MVCs and the feasibility of expanding these roles.

**Results:**

All participants reported benefits from their role whether that was giving or receiving rides. Many accompanied participants reported missing medical appointments prior to joining PIC and being able to avail of the services of a MVC. Volunteer roles varied and ranged from transportation only, help with care coordination and in some cases accompanying the person into their medical visit. A subgroup of volunteers expressed a willingness to take on additional roles during the physician visit following additional training and isolated older adults welcomed the prospect of their assistance.

**Conclusion:**

Our qualitative data indicate that non-family, volunteer MVCs are willing and able to assist older people going to a medical visit. With appropriate training and support, volunteer companions could do much to improve the healthcare experience for those who otherwise would attend alone or would not attend medical visits.

**Supplementary Information:**

The online version contains supplementary material available at 10.1186/s12877-021-02162-5.

## Background

One in five older adults lack a family member, friend or other caregiver to support them as they age [1]. They live alone, often physically and socially isolated and their limited support is unrecognized by health care providers and the community. As our older population grows [2], so too does the prevalence of these so-called “elder orphans”. Awareness of this vulnerable population is growing with the development of screening guidelines and community resources to identify and help these older adults [3] - but much more can be done. Despite the many benefits of being accompanied on a medical visit [4, 5] and strong recommendations supporting being accompanied [6–8], many vulnerable older adults either attend alone or fail to attend [9].

Volunteerism has been found to be very common in the United States. One in four Americans volunteer with an organization and two out of three Americans help their neighbor [10]. Volunteering has many benefits for health. It is predictive of better mental and physical health [11, 12], life satisfaction [13], self-esteem [13, 14], happiness [15, 16], lower depressive symptoms [14, 17], lower psychological distress [13, 14], and mortality and functional inability [14, 15, 18]. As the gap widens between older adults’ need for care and companionship, and the availability of family members to provide that care [19], volunteer companions are a potential solution to bridge the gap.

Volunteer provision of support with eating and drinking, mobilization and therapeutic activities in the in-patient setting has been shown to positively impact patient outcomes [20]. The effects of volunteers in community healthcare related settings are less clear with small studies and often inconsistent effects [21, 22]. Training of volunteers is often not well described and varies considerably between studies and volunteer organizations. Few studies report on volunteers in church groups or community organizations who accompany older adults to medical appointments [22, 23], and there has been little insight into how they view their experiences, or what additional training and organization might be helpful. Using qualitative methods, we examined the experiences of non-family volunteer medical visit companions accompanying older adults to physician visits. We also examined the experiences of the older adults receiving these companionship services. We partnered with a community organization, Partners In Care (PIC), and conducted focus groups with both the volunteers accompanying older adults to physician visits, and the patients who were brought to their visit by a community volunteer. We sought to understand the roles that volunteers are currently playing and learn more about the acceptability of volunteers being involved in the visit itself from the perspectives of both the older adult and the volunteer and also to explore the barriers and facilitators of expanding the availability and roles of medical visit companions.

## Methods

### Study design

Purposeful sampling was used to identify participants for each focus group who had either experience accompanying or being accompanied to a medical visit to ensure that shared common experiences defined the persons in each group. A flexible and structured approach was used, allowing participants to share their experiences [24]. The group moderator’s role was to facilitate rather than direct the discussion to specific topics [25, 26].

### Study population and setting

We partnered with Partners In Care (PIC) [27] a community based organization in Anne Arundel county, Maryland in the eastern United States to recruit focus group participants. PIC provides programs and services that support the independence of older adults using the time and talents of their members, leadership, and staff. PIC has a unique philosophy in which services are provided in exchange for donated time and talents. All members contribute in accordance with their abilities. PIC coordinates over 10,000 rides a year to members, provided by other PIC members, as well as resource navigation, healthy living education, home repairs, and social visits to the homebound. Those with physical or functional limitations also contribute in many ways including writing cards or making phone calls to socially isolated members. Volunteers accompanying members to medical visits are asked to provide a door-to-door service picking up the person at their home, bringing them to their appointment, waiting for them at the providers’ offices, and driving them home again.

### Recruitment

Eligible focus group participants were members of PIC who were at least 50 years old and who had given or received at least one ride to a medical visit in the last 3 months. Participants did not receive monetary compensation for their participation in the focus group; instead, they received volunteer hours in their “PIC bank” that could be “cashed in” for future services. Potential participants received a letter from PIC inviting them to take part in the study. If they expressed interest, an informational leaflet describing the study was mailed to them. A phone call from PIC staff followed to confirm their interest in participation. Eight to 10 members were invited to each focus group. Focus groups followed one of two semi-structured interview guides (supplementary Figs. 1 and 2), one for those who provided companionship during medical visits and one for those who were accompanied to their medical visits. Topics covered with MVCs included their relationship with the PIC and the member(s) whom they were accompanying to medical visits, their history of volunteerism, training received and their roles, expectations and experiences before, during and after the medical visit. Conversely, those who were accompanied were questioned about their experiences of being driven and accompanied on their medical visit. The focus group started by exploring the participant’s history with volunteerism and PIC then moved on to explore what led them to seek a MVC, the difficulties they experience around a medical visit, the tasks for which the MVC provides assistance and their openness and comfort with expanding the role of MVCs. Four focus groups were conducted – two focus groups with 15 participants who accompanied older adults to their visit and two focus groups with 14 persons who were accompanied to their visit by MVCs.

The moderators (a geriatrician and a social worker) had extensive experience in conducting focus groups. Focus groups were held at locations convenient to participants. Before the focus groups began, written informed consent to participate was obtained from all participants. The groups were audio recorded then transcribed verbatim with any identifying information removed during the transcription process. The transcripts were verified for accuracy then stored on a password-protected computer. Audio recordings were erased upon completion of the transcription.

### Data analysis

The data were first analyzed for thematic content specific to accompanying someone or being accompanied to a medical visit using Atlas.ti, a software program used in qualitative data analysis [28, 29]. Both moderators coded the data independently, and then the results were compared. A codebook defining each theme and identifying representative passages from the data was created after reaching coding consensus [30]. The data were analyzed in the same order that it was collected (first MVCs, then those who were accompanied). Discrepancies were resolved through discussion in a process of constant comparative analysis [25]. Interrater agreement between the two coders was greater than 80% for all analyses.

## Results

### Participant characteristics

Forty-eight potential participants were approached for the study and 29 participated, representing a 60% response rate. The main reason given for not participating was “other commitments” (*n* = 15). Many participants (both medical visit companions and accompanied persons) had a strong history of volunteering in their community, schools, civic organizations and churches or had dedicated their work life to working with under resourced communities. One participant indicated that they had spent their “career, about 30 years, helping run a non-profit” in the area. Another participant shared that they worked in the public service, doing advocacy work on behalf of vulnerable people including troubled youth, veterans and the elderly. For many participants joining a volunteer organization like Partners In Care was a natural progression of their interest and experience with service.

Descriptive information about the MVC and patient samples is provided in Table 1. Medical visit companions ranged in age from 62 to 85 years. Most were Caucasian (93.3%), female (73.3%), and retired (80%). Most MVCs (80%) reported having a bachelor’s degree or higher. People receiving rides had a similar age range, and again most were Caucasian (71%), female (86%), and retired (86%). Most people who were accompanied reported having some college education (71%). Two major themes emerged from the data: (1) the benefits of being/having a volunteer companion and (2) the need for additional training for those participants who desired more engagement during the medical visits.

**Table 1:** Characteristics of Volunteer Companions and Accompanied Persons

	Volunteer Companions ***N*** = 15	Accompanied Persons ***N*** = 14
**Age (years), mean (SD)**	72 (5.9)	76 (7.0)
**Race**, ***n*** **(%)**		
African American	1 (6.7)	3 (21.4)
Caucasian	14 (93.3)	10 (71.4)
Asian	0 (0.0)	1 (7.1)
**Gender,** ***n*** **(%)**		
Female	11 (73.3)	12 (85.7)
**Rides provided in last 3 months,** ***n*** **(%)**		
1–3	0 (0.0)	2 (15.4)
4–7	1 (6.7)	5 (38.5)
More than 8	14 (93.3)	6 (46.2)
**Ride location,** ***n*** **(%)**		
Primary Care Doctor	5 (27.8)	4 (22.2)
Specialist	8 (44.4)	7 (38.9)
Other healthcare appointment (e.g. laboratory)	4 (22.2)	2 (11.1)
Other places (pharmacy)	1 (5.6)	1 (5.6)
**Education,** ***n*** **(%)**		
HS or less	0 (0.0)	5 (35.7)
Bachelor’s or less	9 (60.0)	8 (57.1)
Higher degree	6 (40.0)	1 (7.1)
**Relationship status,** ***n*** **(%)**		
Single	1 (6.7)	1 (7.1)
Married	8 (53.3)	0 (0.0)
Separated/divorced	4 (26.7)	5 (35.7)
Widowed	2 (13.3)	8 (57.1)
**Employment,** ***n*** **(%)**		
Part-time	2 (13.3)	0 (0.0)
Receiving disability	0 (0.0)	2 (14.3)
Retired	12 (80)	12 (85.7)
Not currently employed	1 (6.7)	0 (0.0)
**Household income,** ***n*** **(%)**		
Less than 50,000	3 (20.0)	13 (92.9)
$50,000 - $100,000	7 (46.7)	0 (0.0)
$100,000 +	3 (20.0)	0 (0.0)
Prefer not to answer	5 (33.3)	1 (7.1)

### Benefits of being/having a companion

#### Volunteerism

Participants recounted how much volunteering meant to them and the impact PIC has on their lives. Several mentioned how volunteering as a companion had benefited their own health. One participant stated:

*“The thing that was a particular advantage to me was that I wanted to get over my fear of being in doctors’ offices. My blood pressure would always go up … but that’s helped me .. I walk into all kinds of doctors’ offices now so I feel a lot more relaxed going in my own visits” (Medical Visit Companion 10).*

Another benefit participants from both groups shared was the enriching experience of meeting amazing people that they otherwise never would have met. One driver learned that his father and the husband of the person he was accompanying served at the same base in England during World War II and was delighted to exchange stories.

#### Experience of being accompanied

Those accompanied to medical visits outlined details of the many benefits they received because of the generosity of the volunteers. Many stated that they would be unable to go to their medical appointments at all without the volunteer drivers and two participants reported skipping appointments before joining PIC:

*“I couldn’t get to the doctor’s appointment any other way” (Accompanied Person 5).*

Although some people had family who could take them to medical visits if needed, many described their children as being unable to get time off work. One lady described:

“*If I don’t give him 3 or 4 weeks’ notice he’s not taking me. Because he has to ask off work. He means it 100%. I found out the hard way. I missed my appointment” (Accompanied Person 6).*

The presence of a volunteer companion relieved a lot of the anxiety around visits with patients describing the security they felt knowing that they would not be alone from the time they left their home until they returned.

“*I’m not dropped off. I know he’s there. When I come out, he’s sitting right there…it is important” (Accompanied Person 8).*

Not only were they able to get their appointments, but they were also on time – avoiding fees or having to reschedule an appointment. One person described two particular volunteers who coordinate with each other to make sure one of them can always take her to her appointments.

Going to a visit with a volunteer companion was described by one lady as “like going out with your friend”. Even long wait times at some clinics did not change the demeanor of the volunteers.

“S*ometimes we have to wait as long as 4 hours before we get to leave. And that’s a long time to have (someone) wait. But they’ve always been so patient and understanding*.” *(Accompanied Person 12)*

Patients described many other advantages such as not having to endure long waits for mobility services to bring them home and being able to stop at the pharmacy or grocery store if needed on the way home. Some of the drivers shared that for some people they accompany, going to the doctor may be one of their few opportunities to socialize. Many volunteers responded by making the drive home an opportunity for an outing. A participant described:

*“I had said it would be good to go to lunch .. after we went to a visit and I thought that I would enjoy that, they would enjoy that.”(Medical Visit Companion 4)*

Ride recipients were particularly grateful for help with certain tasks such as paperwork completed in the waiting room.

*“Doctor’s offices are now handing you clipboards...and you have to fill out all this stuff, it’s overwhelming if you’re not feeling well…..”*.” *(Accompanied Person 14)*

#### Joining the physician visit

Two distinct groups of companions emerged, those who welcomed more involvement in the actual medical visit and those who were content with providing transportation only.

Companions who described their experiences of providing additional support during the medical visit explained that their role changed slowly over time from primarily providing transportation to accompanying the person into the visit. One participant described how

“*I had a nice lady ask me once could I go in with her because ... she doesn’t understand everything her doctor says. And sure enough she had had some significant health issues and wasn’t picking up what he was laying down. So on the way home we just discussed more and I offered to talk to her daughter over the phone and she said, no, I think I understand now*”. *(Medical Visit Companion 14)*

Many volunteers were content in their door-to-door transport role and expressed concerns about additional involvement, feeling that they may become emotionally entangled or unsure how to deal with confidential information. As one driver stated:

“*It’s very, very different if you’ve got somebody you just met and will probably never see again and you are barging into their life and then if that person lives alone, you’ve got nowhere to turn the data over afterwards. I think that borders on really can very well be intrusive rather than helpful.” (Medical Visit Companion 3)*

Others felt that being present in the visit could prompt providers to address them rather than the patient. A participant who experienced this described:

“… *they began to talk to me and not to her. And I realized that she was so grateful this was great because she didn’t have to pay attention or focus. But they also talked too fast ... I’m pretty sure they didn’t talk to me the way they talked to her. But it was so much easier because they could just go “blrrpp” to me and then they knew that I would translate it*.” *(Medical Visit Companion 5)*

### Need for additional training or support

Many companions indicated that they provided additional assistance, including help with filling out forms, assisting with scheduling follow up visits, taking the patient to pick up new prescriptions from the pharmacy and relaying information to other family members. Many participants had concerns about patient confidentiality and had a poor understanding of privacy rules. Subthemes helped to identify opportunities for additional training.

All of the people who reported accompanying people into a visit expressed a wish for additional training to better understand the responsibilities of their expanded role. *“…but you do something when it seems to make common sense or it’s something that would help a person you know so if there’s more information available on working with people in medical situations, sure. I think that a lot of us would be interested in it.” (Medical Visit Companion 6).*

### Preparing for the visit

Participants reported that preparing for the visit starts as soon as a person requests a ride and a driver accepts. Drivers took the responsibility of getting the patient to their appointment on time very seriously, and prepared in advance to avoid unnecessary stress on the patient. Drivers discussed using GPS or MapQuest, and often doing a dry run a few days beforehand to ensure they know where they are going. As one participant stated

“… *I try to get them there 10 or 15 minutes before the appointment. So they don’t feel stressed about having to rush to get there… if you’re late, they’re in trouble, they can lose money, they can be charged for not showing up on time*.” *(Medical Visit Companion 10)*

Some drivers felt comfortable asking patients if they had their insurance card, medication list and referral letter with them before they left their house and described times where they had helped to find these items before leaving the house.

### Advocacy

Although Partners In Care recruited their volunteers to only provide rides and counseled them about getting overly involved with the people they drive to medical appointments, some volunteers indicated that they chose to get more involved during the medical visits for the patient’s benefit. Many participants we spoke with had been volunteer companions for many years and have become attached to particular people. One participant shared that the person she accompanies will only make appointments on a day that she can bring her as she doesn’t want anyone else to take her. Some drivers who have long relationships with a person mentioned that over time they noticed that they had to step in to help more, including going in the consultation room:

“… *I have to go in with her because she can’t understand what he’s saying to her. And in fact, I get a hug from the doctor as well*.” *(Medical Visit Companion 11)*

### Accompanying someone with physical difficulties

Participants expressed concern about knowing how best to accompany someone with mobility problems. Although most people reported a willingness to drive anyone they could in their car, they were concerned that they would not know what to do if the person needed help with equipment or transferring in and out of the car. One driver stated

“*Even though I know I’m not supposed to help somebody [with gait problems], I would feel much more confident if I had training to do that. … you don’t know whether they actually do need or will need help, but just in case it would be nice if you knew what to do if they did stumble or if they’re slipping, something they don’t normally do, like it’s icy or raining*.” *(Medical Visit Companion 10)*

### Confidentiality

Attitudes and behaviors around discussing health information varied among participants. Some did not want to discuss anything with a companion especially one that they were meeting for the first time. A number of older adults discussed health issues before or after a trip including sharing concerns about the upcoming visit or expressing relief, concern or other emotions after the visit. Health information was often shared in the waiting room or at check in when the companion provided assistance to the older adult completing intake forms. In most cases where health information was shared, a rapport had been built between the two parties over several trips. Many participants were confused about confidentiality issues with some believing that if they learned any personal details about the medical visit they would be violating HIPPA rules. One driver indicated that they did not feel comfortable providing assistance if they learnt confidential information about the person they were accompanying. Others were uncomfortable assisting with form filling:

“[*the receptionist wanted] me to fill out his paperwork, and again like she was saying it was, to me it was a private matter and she had the right to fill out his paperwork more so than I did … so I declined to.”(Medical Visit Companion 13)*

Another participant remarked in response, *“…you start crossing some lines with HIPPA and few other regulations*”. *(Medical Visit Companion 8)*

*What to do with what you hear during the visit.*

Participants felt that if they learned more about the medical condition of the patient, they would feel responsible and compelled to do something about it. There was also a suggestion that they would become emotionally involved and that was not the direction they wanted to go in. They felt that the emotional well-being of the person they accompanied was not the volunteer’s responsibility:

*“No because I typically don’t ever see them again and I really don’t want to be responsible for their continuing care. I think that’s really dangerous for me to get involved that closely”.*

Concerns were raised about what to do if the volunteer knew that the person they were accompanying was not going to follow the healthcare provider’s instructions. One participant indicated that a person she accompanied once told her after a visit

“… *they’ve given me all these meds and, but I’m not gonna take them, I throw them away. I would like to, what I am supposed to do with that information*?” *(Medical Visit Companion 4)*

Partners In Care encouraged volunteers to report any such issues to them. This removed the responsibility from the volunteers and many people described this as very reassuring, however, it is clear that further training is necessary around confidentiality of medical information,

## Discussion

We examined the experiences of 29 members of a community organization who either gave or received rides to medical visits, by conducting focus groups and studying the themes that emerged. Many ride recipients received help with other tasks such as remembering what to bring to their appointment, form filling, scheduling, advocacy, providing collateral histories and much more. Both parties clearly enjoy and benefit from the medical visit companion program, and although it was not part of their expected role, for people they accompanied with more significant health problems many drivers clearly provided a care coordinator role.

One important finding was the different levels of comfort participants had when involved in the medical visit itself. Many people who volunteered to give rides saw the visit as a confidential area they should not enter. However, many of these same people did not see any issue with viewing referral letters, insurance documents or helping to fill out medical history forms in the waiting room. The confusion that volunteer MVCs expressed around confidentiality and HIPAA are issues that could easily be addressed with training. It was clear that many volunteers are willing to do much more than merely drive someone from door-to-door. The acceptability of volunteer drivers participating in medical visits also varied for the person receiving the ride. There was a clear distinction between people who appreciated the volunteers taking them to their appointment but who had family members that they would call on if they were going to a visit they considered “serious”, and people who had no family or close friends to rely on who would be incredibly grateful if a volunteer would accompany them into their medical visit. Similar issues have been reported in befriending studies in the mental health literature where volunteers report their befriending experience positively but express confusion about roles and express varying levels of comfort with different levels of commitment [31]. It was clear was that many volunteers are willing to do much more than merely drive someone from door-to-door. The acceptability of volunteer drivers participating in medical visits also varied for the person receiving the ride. There was a clear distinction between people who appreciated the volunteers taking them to their appointment but who had family members that they would call on if they were going to a visit they considered “serious”, and people who had no family or close friends to rely on who would be incredibly grateful if a volunteer would accompany them into their medical visit. Many of these isolated older adults reported missing or not being able to attend medical visits prior to joining PIC and gaining a MVC. Larger studies are needed to examine the effect of MVCs on attendance at medical visits perhaps targeting older adults who frequently miss appointments and linking them with a MVC to increase availability and awareness of the service and its potential benefits.

It was also clear that in many cases a rapport developed over time between the older adult attending the visit and the person accompanying them. As the relationship, and sometimes friendship developed the comfort level increased with sharing healthcare information, asking that person to accompany them again or help with additional tasks. Systematic reviews confirm the absence of a typical volunteer but instead highlight the need for volunteers from a wide variety of backgrounds to act in these companion roles [32, 33].

As healthcare becomes more complex to navigate, care coordinators are becoming increasingly important. Many of the volunteers we spoke with performed care coordination roles without fully understanding that.. As the gap widens between older Americans’ need for care and the availability of family members to provide that care, the availability of volunteers willing to step into these roles becomes increasingly valuable. It is clear from speaking with the members of Partners In Care that there are many willing volunteers in our communities ready and able to help our vulnerable older population. We propose a tiered system driven by the needs and wants of the patients, with some volunteers providing a door-to-door ride service, while others provide some forms of care coordination, and a small number of volunteers accompanying older adults into physician visits. Studies examining the role of volunteer companions in the community setting show mixed results with positive effects of befriending in the mental health setting [31] but unclear benefits when used to target social isolation [22, 34] or address healthcare utilization. Training for these volunteers is inconsistent and may contribute to the different effects observed in different studies. Our volunteers felt that they had received adequate training for their transportation role but additional training would be required to allow them to comfortably expand their roles. Guided by the findings of our work, we feel that tailored training on the roles and expectations for these volunteers could be provided at the community level and support provided as needed to ensure a good experience for all and allow effective change.

The strengths of this study include the unique population of experienced community volunteers and the fact that we were able to capture the perspectives of both the people who accompany and are accompanied to medical visits. The sample was however, homogeneous, self-selected, educated and derived from a single community organization in suburban Maryland and the generalizability of our findings need to be studied in other diverse populations.

## Conclusion

As more and more older people lack an available family member to accompany them to medical visits, many community organizations, churches and senior centers are ready and willing to help. With appropriate training and support, volunteers can fill this important gap for many older adults and ensure that someone is always available to accompany older adults to their health care visits.

## Supplementary Information

**Additional file 1:.** Semi-structured interview guide: Persons accompanied to medical visit

**Additional file 2:.** Semi-structured interview guide: Persons who accompany older adults to medical visits

## Data Availability

The datasets used and/or analysed during the current study are available from the corresponding author on reasonable request.

## Abbreviations

PIC: Partners In Care
MVC: Medical Visit Companions
AARP: American Association of Retired Persons
NIA: National Institute on Aging
HIPAA: Health Insurance Portability and Accountability Act
