# Supplementary material for: Can volunteer medical visit companions support older adults in the United States?
Source: BMC Geriatr. 2021 Apr 16;21:253. doi: 10.1186/s12877-021-02162-5 (PMC8052762; doi:10.1186/s12877-021-02162-5)
Supplement: Supplementary file 2 — Additional file 2:. Semi-structured interview guide: Persons who accompany older adults to medical visits [file 12877_2021_2162_MOESM2_ESM.pdf]

Semi-structured interview guide: Persons who accompany older adults to medical visits

Relationship with Partners in Care

1. How long have you been a member of PIC?
2. Approximately how many hours per week do you volunteer?
3. What is your history of volunteering? Have you ever volunteered before? Doing what? What was that commitment?
4. What are the advantages and disadvantages of being a member (to the recipient AND to yourself)?
5. What does someone need to have or to do to be a good volunteer in PIC?
    a. What would the description of a care provider look like?
    b. What do you think are the characteristics of a good volunteer?
6. How did you become involved with PIC?
7. In what ways do you volunteer with PIC?
8. What are some areas of improvement in the program?
9. Do you feel like you could volunteer more now or in the future? If so, what are some of the reasons/factors that would allow you to volunteer more?
10. Do you feel like you want to volunteer less now or in the future?
11. What made you request a ride?
12. Are there particular places you request rides to? – pharmacy, medical visit, grocery store?
13. How often do you request rides?
14. Does the same volunteer pick you up most times?
15. Where do you sit? Front seat? Back seat?
16. What kind of training, if any, did you receive about receiving rides?
17. When the person brings you to your medical visit do they come into the office with you?
18. If yes, do they come into the consultation with you?
19. If yes, is that because you ask them to, they volunteer or it is just assumed?
20. If not, why not?

Bringing someone to a visit

1. How do you prepare before you go to a medical visit?
    - Write down questions, find insurance cards etc.
2. When you are picked up does the person ask you anything about your readiness for the visit before you leave the house?
    - Do you have your medications? Are there any test results you need to bring? Do you have your insurance cards?
3. Are these reminders helpful or if they are not being asked currently would you find them helpful?
4. Do you discuss your upcoming visit during the car ride?
    - What do you discuss? Where you are going, the provider, your health conditions, your worries, your questions.

5. Would you find it helpful to talk out your questions on the ride or do you prefer to keep them to yourself?

The visit itself

General questions

6. Do you find it useful to have someone with you at a medical visit?
   Why or why not?
7. Do you introduce the person who is with you and explain who they are?
8. Do you take notes? If no, would you like the person accompanying you to take notes?
9. If the ride partner accompanies you would you prefer for them to join in the discussion or just listen?
10. Do you normally understand everything the doctor says?
11. If the ride volunteer doesn't come into the visit with you do you think you would benefit if they did?
    If so, how?

After the visit

12. What benefits, if any, do you get from having a ride partner?
13. If a ride volunteer joins you in the visit what benefit, if any do you get from being accompanied to a medical consultation?
14. On the ride home do you discuss the visit?
15. On the ride home do you discuss the visit?
    - If so, what specific details of the visit are discussed?
16. If there are changes to medications do you stop at the pharmacy on the way home?
17. Does the same person bring you to follow up visits?
18. Do they ever help you to arrange tests, follow up appointments etc.?
19. If the person who gives you a ride doesn't accompany you into the visit, would you consider asking them to do this in the future?
20. If you did not have the service, what would you do?
    a. What is the biggest advantage?
    b. What is the biggest disadvantage?
21. Do you have any confidentiality concerns?
